# Fournier gangrene presenting in a patient with undiagnosed rectal adenocarcinoma: a case report

**DOI:** 10.1186/1757-1626-2-9136

**Published:** 2009-12-03

**Authors:** Mohammd Kazem Moslemi, Mohammad Ali Sadighi Gilani, Ali Akbar Moslemi, Ali Arabshahi

**Affiliations:** 1 Department of Urology, Kamkar Hospital, Qom Medical Sciences University, School of Medicine, Bajak Ave, Qom, Iran; 2 Department of Urology, Shariati Hospital, Tehran Medical Sciences University, School of Medicine, Kargar Shomali Ave, Tehran, Iran; 3 Department of Mechanics, South Methodist University, Dallas, Texas, USA; 4 Department of Health, Kamkar Hospital, Qom Medical Sciences University, School of Medicine, Bajak Ave, Qom, Iran

## Abstract

**Introduction:**

Fournier gangrene is a rare necrotising fascitis of the perineum and genitals caused by a mixture of aerobic and anaerobic microorganisms. The first case was described by Baurienne in 1764 but the condition was named by Fournier in 1883 who reported the cases of five men with the condition with no apparent etiology. Infection most commonly arises from the skin, urethra, or rectal regions. Despite appropriate therapy, mortality in this disease is still high. We report a case of a low rectal malignancy presenting as Fournier gangrene. This case report serves to highlight an extremely unusual presentation of rectal cancer, a common surgical pathology.

**Case presentation:**

The patient is a 48 years old Afghanian male that admitted with Fournier gangrene. In the course of medical and surgical treatment the presence of extensive rectal adenocarcinoma was discovered. After partial recovery, standard loop colostomy was inserted. Skin grafting of necrotic areas was performed and systemic rectal cancer chemotherapy initiated after full stabilization.

**Conclusion:**

Fournier gangrene is an uncommon but life threatening condition with high associated mortality and morbidity. Usually there is an underlying cause for the development of Fournier gangrene, that if addressed correctly, can lead to a good outcome. Early diagnosis and treatment decrease the morbidity and mortality of this life threatening condition. Good management is based on aggressive debridement, broad spectrum antibiotics and intensive supportive care.

## Case presentation

The patient is a 48-year-old Afghanian male that referred to our center due to swelling, redness and tenderness of external genitalia, that found incidentally in the investigation of cachexia and weigt loss. He revealed no genitourinary or gastrointestinal complaints before admission. After admission full physical examination performed.

On examination, patient was cachectic with BMI of 18 kg/m2. He was afebrile and initial hematological investigations revealed only a moderate leucocytosis (WBC count, 18000 Cells/HPF) with mild anemia (Hg 11 g/dl). External genitalia and perianal examination revealed skin necrosis, pussy drainage and subcutaneous crepitations, in addition to above mentioned signs.

**Figure 1:**
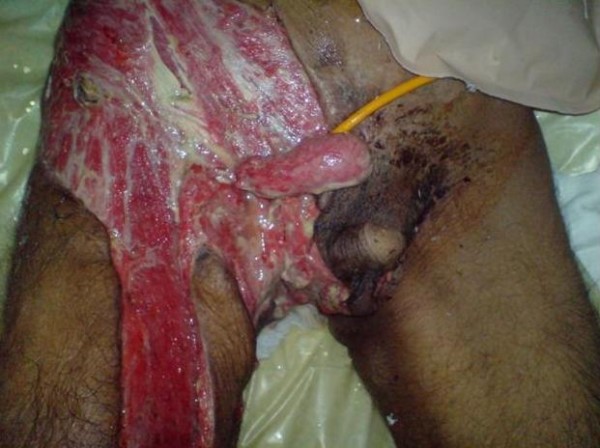
**The patient after full recovery and 3 day after colostomy insertion**.

**Figure 2:**
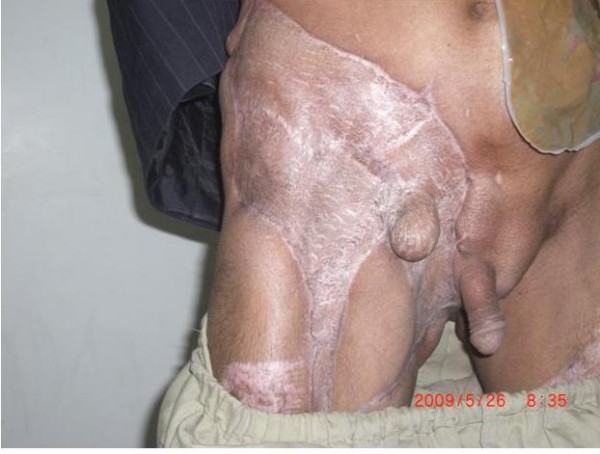
**The patient 6 month after skin grafting**.

Urgent and vigorous intravenous triple antibiotic therapy with Ceftriaxone (2 grams Q 12 h), Metronidazole (500 mg Q 8 h) and Amikacin (500 mg Q 12 h) was commenced. He was taken to operating room, multiple times, and every time vigorous debridement performed. A thorough washout was performed. Following acute management a thorough examination including digital rectal examination was performed which detected an extensive rectal mass with extension of necrotic points to the anal area. Punch anal biopsy performed and sent for histological analysis, which showed rectal adenocarcinoma. The zone of necrosis covered a large area from external genital area to the inner aspect of right thigh to the right side of the abdomen (Figure 1). A CT scan revealed a large rectal tumor with pelvic adenopathies. In the course of surgical management the patient received multiple units of packed RBC due to intermittent acute bleeding after each surgical debridement and also anemia of chronic disease-underlying malignancy. He also received regular infusion of parenteral nutrition. After partial stabilization, a standard left loop colostomy performed (Figure 1) to prevent an impending large bowel obstruction and to promote healing of the perineum. After full stabilization, skin dermal grafting for full coverage of involved areas performed (Figure 2).

Systemic chemotherapy for the treatment of underlying rectal adenocarcinoma initiated, the patient was well one year after first visit.

## Discussion

Fournier gangrene is a rapidly progressive necrotizing fasciitis of the perineum and external genital organs. The first documented clinical picture of the disease was made by Fournier in 1883. Its epidemiology has changed and is much different from the origin. It affecting mostly men between 50 and 60 years of age. This disease also affects females. It is uncommon in pediatric age group and little is known about the disease in the newborn period and infancy [1]. It is secondary to polymicrobial infection by aerobic and anaerobic bacteria with a synergistic action. Candida albicans has also been reported as the primary microorganism [2]. The most commonly isolated microorganism in both male and female patients was E coli [3]. The aetiology is identified in 95% of cases. The source of infection is either cutaneous, urogenital or colorectal areas. Predisposing factors, such as age, diabetes, alcoholism, malnutrition, and immunosupression, are often present in affected patients. In one study Diabetes mellitus was the most common associated disease in these patients, however, it was not related to a statistically significant worst prognosis [4]. On the basis of Cakmak study on the 65 cases the most common etiology was hemorrhoidectomy in male and perianal abscess in female patients [3]. In a series of 45 patients with fournier gangrene, Basoglu et al reported that the most common etiology was perirectal abscess followed by scrotal carbuncle and thrombosed hemorrhoid [5]. This series, which is among the largest ever published, did not identify colorectal carcinoma as an etiology. Korkout et al reported that the etiology was identifiable in 95% of cases, diabetes mellitus being a predisposing factor in 55.6% [6]. Our patient notably had no such commonly described etiology or predisposing factor leading to the conclusion that gangrenous disease was a manifestation of low rectal cancer. A thorough literary review revealed only four reported cases of Fournier gangrene resulting from colorectal carcinoma during the last 30 years [7-10]. The carcinomas described in those reports typically comprised a perforating adenocarcinoma of the sigmoid colon, leading us to believe that Fournier gangrene is an extremely unusual presentation of rectal cancer. Urgent and aggressive treatment is essential to ensure the patients survival. Treatment consists of restoration the fluid and electrolyte balance and broad spectrum antibiotic therapy rapidly followed by surgical debridement. However, the mortality remains high, about 20 to 80%, frequently, due to delayed diagnosis and management. Patients who survive the infection require reconstructive surgery with sometimes marked sequelae related to the extent of fasciitis and debridement [11]. An association between urethral obstruction associated with strictures and extravasation and instrumentation has been well documented. Predisposing factors include diabetes mellitus, local trauma, paraphimosis, periurethral extravasation of urine, perirectal or perianal infections, and surgery such as circumcision or herniorrhaphy. In cases originating in the genitalia, the infecting bacteria probably pass through Bucks fascia of the penis and spread along the dartos fascia of the scrotum and penis, Colles fascia of the perineum, and scarpas fascia of the anterior abdominal wall. The Infection commonly starts as cellulitis. Early on the involved area is swollen, erythematous, and tender as the infection begins to involve the deep fascia. Pain is prominent, and fever and systemic toxicity are marked. Because crepitus is often an early finding, a plain film of the abdomen may be helpful in identifying air. The mortality rate averages approximately 20%, but ranges from 7% to 75%. This is similar to our in-hospital mortality rate. Higher mortality rates are found in diabetics, alcoholics, and those with colorectal sources of infection [12].

In a large population based epidemiologic study, 1641 males and 39 females with fournier gangrene identified. Cases represented less than 0.02% of hospital admissions. The overall incidence was 1.6/100,000 males, which peaked in males who were 50 to 79 years old. The overall case fatality rate was 7.5% [13]. For predicting prognosis, Fournier gangrene severity index presented [14]. Using a Fournier gangrene severity index threshold of 9 (sensitivity 71.4%, specifity 90%) there was a 96% survival rate in patients with a Fournier gangrene severity index of less than 9 and a 46% mortality rate in those with a Fournier gangrene severity index of 9 or greater (p = 0.001, OR 22,95% CI 3.5-139.7) [14].

## Conclusion

Necrotizing fascitis of the perineum and genitalia is a severe condition with a high morbidity and mortality. It seems that there are no major differences between male and female patients in the characteristics of the condition [3]. Good management is based on aggressive debridement, broad spectrum antibiotics and intensive supportive care [15]. In the early stages of the disease, before necrotic lesions occur, a final diagnosis might be difficult. Hyperbaric oxygen therapy and reconstructive procedures may be needed in the course of treatment process [16]. The fournier gangrene severity index remains an objective and simple method to quantify the extent of metabolic aberration at presentation in patients with founier gangrene. A Fournier gangrene severity index threshold value of 9 is sensitive and specific for predicting mortality in this patient population [14]. The patients age, the presence of systemic risk factors, especially cancer, a urological source of infection and the extent of disease have impact on the prognosis of Fournier gangrene [4]. Although colorectal carcinoma has previously been reported as a cause of Fournier gangrene it remains an extremely rare phenomenon.

## Consent

Written informed consent was obtained from the patient for publication of this case report and accompanying images. A copy of the written consent is available for review by the Editor-in-Chief of this journal.

## Competing interests

The authors declare that they have no competing interests.

## Authors' contributions

MMK is the corresponding author and responsible for the surgery, SGMA assisted in surgery and editing the manuscript, MAA and AA were major contributors in writing and editing the manuscript. All authors read and approved the final manuscript.
